# Measles and Rubella during COVID-19 Pandemic: Future Challenges in Japan

**DOI:** 10.3390/ijerph18010009

**Published:** 2020-12-22

**Authors:** Kazuki Shimizu, Ayaka Teshima, Hiromi Mase

**Affiliations:** 1 Department of Health Policy, London School of Economics and Political Science, Cowdray House, Houghton Street, London WC2A 2AE, UK; 2 Faculty of Public Health and Policy, London School of Hygiene and Tropical Medicine, Keppel Street, London WC1E 7HT, UK; 3 Graduate School of Medicine, Hokkaido University, Kita 15 Nishi 7, Kita-ku, Sapporo 060-8638, Japan; 4 Faculty of Medicine, School of PUBLIC Health, Imperial College London, St Mary’s Campus, Norfolk Place, London W2 1PG, UK; ayaka.teshima20@imperial.ac.uk; 5 Department of Epidemiology and Public Health, Institute of Epidemiology and Health Care, University College London, 1-19 Torrington Place, London EC1E 7HB, UK; hiromi.mase.19@ucl.ac.uk

**Keywords:** infectious disease policy, measles, rubella, importation, hygiene, immunization, health service delivery, mass gathering, COVID-19, Japan

## Abstract

The coronavirus disease 2019 (COVID-19) pandemic has significantly impacted essential health services. Simultaneously, it has created opportunities for citizens to raise awareness of personal hygiene, mask wearing, and other preventive measures. This brief report aims to clarify the epidemiological trends of measles and rubella in Japan and to explore future challenges for controlling these diseases during and after the COVID-19 pandemic. Although Japan eliminated measles in 2015, the number of measles patients has gradually increased since then, and reached 744 in 2019. In the 2010s, Japan experienced two large rubella epidemics, and the majority of the patients were reported in Tokyo and other metropolitan areas. While the transmission of measles and rubella seems to be suppressed during the COVID-19 pandemic, closing the gap in routine childhood vaccination will be challenging in any country. Moreover, supplementary immunization campaigns for adults have also been disrupted, and they must be invigorated. While the pandemic has a devastating effect on a global scale, it should be utilized as a good opportunity to regain faith in vaccines, implement an evidence-based vaccination policy, and strengthen international cooperation.

## 1. Introduction

The coronavirus disease 2019 (COVID-19) has developed into a pandemic, and it significantly impacts the maintenance of essential health services, including cancer care and elective surgeries [1,2]. The delayed implementation of childhood vaccines will be expected to significantly impact the disease burden of vaccine-preventable diseases (VPDs) among children [3]. At the same time, the positive effects brought by the COVID-19 pandemic need to be fairly argued. The high commitment of citizens’ practice to hand washing and personal hygiene and raised awareness of other preventive measures have brought windows of opportunity not only to decrease pediatric admissions due to respiratory diseases [4], but to incorporate public health science into public policies.

While Japan has mitigated the impact of COVID-19 on the health of its population, negative impacts on health services, along with issues in health system capacity, health communication, and governance, have been reported, and protecting the vulnerable population is a critical agenda [5,6,7,8,9,10,11]. It must be noted that these issues have elicited a high commitment of citizens in Japan to health promotion campaigns and policy measures [12,13]. As of today, to what extent the COVID-19 pandemic has worked both positively and negatively for controlling other infectious diseases has not been revealed in the Japanese context. To complement this point, this brief report aims to clarify the epidemiological evolution of measles and rubella in Japan, as these diseases have been recognized as crucial challenges in Japan’s vaccination policy, and are potential health threats for the upcoming Tokyo Summer Olympics and Paralympic Games in 2021 [14,15]. We also explore future challenges for controlling measles and rubella during and after the COVID-19 pandemic, a thought that is becoming a global agenda.

## 2. Materials and Methods

We conducted a descriptive epidemiological investigation of vaccine-preventable diseases. We included measles and rubella in our investigation, comparing the number of reported cases and incidence of measles and rubella in Tokyo with those in the whole of Japan between 2016 and 2020. The breakdown of reported cases, by week, acquired place, age group, and vaccination history was extracted from the Infectious Diseases Weekly Report published by the National Institute of Infectious Diseases [16,17,18]. Weekly reported cases in Tokyo were extracted from the Tokyo Metropolitan Infectious Disease Surveillance Center [19]. These yielded the incidence per million people in Tokyo and the whole of Japan, and the proportion of imported cases. Then, we identified ongoing potential challenges around measles and rubella caused by the COVID-19 pandemic, and explored implications for the future vaccination policy, which will be applied at both national and global levels. Because this study analyzed publicly available, anonymized secondary data, ethical approval by an institutional review board was not required.

## 3. Results

### 3.1. Epidemiological Trends of Measles and Rubella in Tokyo and All of Japan, 2016–2020

**Figure 1:**
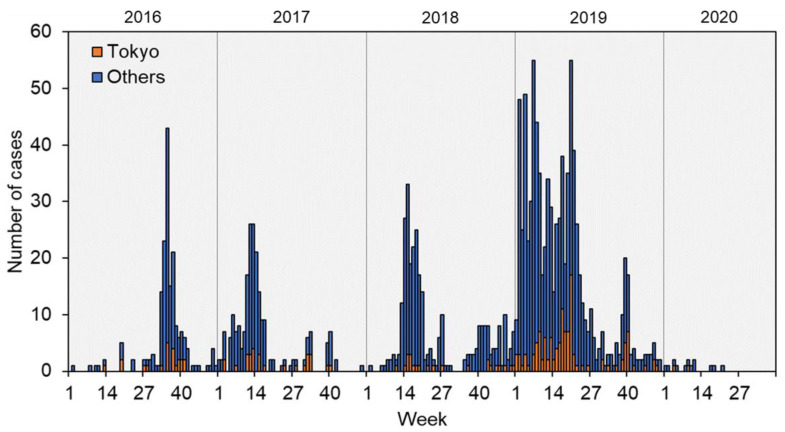
Epidemiological trend of measles in Tokyo and all of Japan, by week, in 2016–2020. The orange represents the number of reported measles cases in Tokyo, while the blue indicates reported measles cases outside Tokyo. In 2020, only weeks 1–39 are shown due to the availability of data.

Figure 1 presents the epidemiological evolution of measles reported in Tokyo and the whole of Japan. During this period, Japan was continually recording more than 100 measles cases per year. The number of yearly reported cases has gradually increased from 159 in 2016 to 744 in 2019; however, the number of measles cases is limited to 12 as of September 2020.

While Tokyo recorded higher numbers of measles cases per population than those in the whole of Japan in 2016, 2017, and 2019, opposite trends were observed in 2018 (Table 1). This is partly due to a large measles outbreak in Okinawa, as previously reported elsewhere [20,21,22].

**Figure 2:**
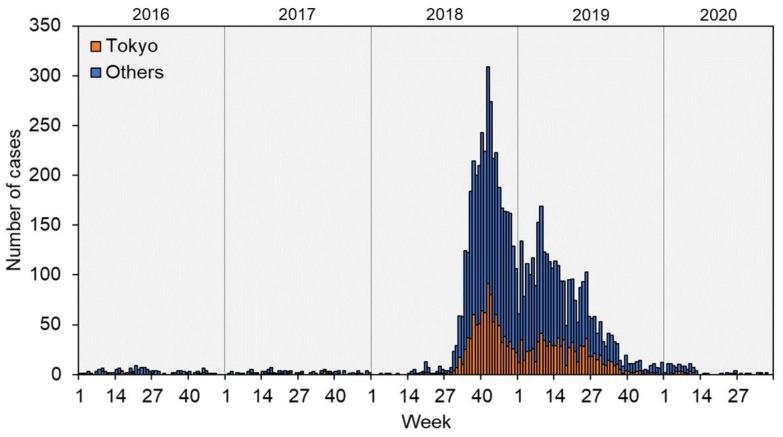
Epidemiological trend of rubella in Tokyo and all of Japan, by week, in 2016–2020. The orange represents the number of reported measles cases in Tokyo, while the blue indicates reported rubella cases outside Tokyo. In 2020, only weeks 1–39 are shown due to the availability of data.

The epidemiological trend of the rubella epidemic in Japan in 2016–2020 is shown in Figure 2. After Japan experienced a large rubella epidemic from 2012 to 2014 [23,24], the yearly reported rubella cases decreased to fewer than 100 in 2017; however, in the summer of 2018, the number of rubella patients peaked at more than 300 cases per week. In 2018 and 2019, the cumulative number of rubella cases amounted to 2917 and 2306, respectively, and a large number of cases were reported from Tokyo (947 in 2018 and 860 in 2019). While Japan recorded around five rubella cases per week in weeks 1–12 in 2020, the number decreased significantly to zero to three per week afterwards. This decrease coincided with periods of increasing health promotion campaigns and the state of emergency because of COVID-19 in April–May [7].

Tokyo consistently recorded higher numbers of rubella cases per million population during the study period. While numbers were limited to fewer than 2 in 2016 and 2017, they skyrocketed to over 60 in 2018–2019, and they were much higher than those in the rest of Japan (Table 2).

### 3.2. Cases Aggregated by Acquired Places

Table 3 presents the yearly number of measles patients aggregated by the place where they acquired the disease. Domestically infected cases accounted for the majority of total patient numbers, while some measles outbreaks occurred as a result of imported measles cases [20,21,22,25,26,27].

While the importation of measles was critically featured in Japan’s health emergency preparedness [14,15,26], both domestically infected cases and cases with unspecified/unknown origin were challenges in the rubella epidemic from 2016 to 2020 (Table 4). Since 2018, more than 20% of all cases have been classified as “unspecified” or “unknown,” suggesting difficulties with contact tracing.

### 3.3. Cases Aggregated by Age and Vaccination History

The reported measles cases by age group and vaccination history are presented in Table 5. Before the COVID-19 pandemic, less than 20% of all cases were reported among those aged 0–9, and cases that had received at least one dose of a vaccine accounted for less than 40%.

The reported rubella cases by age group and vaccination history are presented in Table 6. In 2016–2017, around 20% of all rubella cases were reported among children aged 0–9. This tendency dramatically changed in 2018–2019, when more than 60% of all cases were observed in males aged 20–49. While the reported number is limited in 2020, men aged 40–49 accounted for 26% of all cases. In terms of vaccination history, less than 10% of patients had been vaccinated for rubella at least one time in 2018–2019, and around 65% of all cases had an unknown vaccination history in 2018–2020.

## 4. Discussion

### 4.1. Evolution of Measles and Rubella in Japan and the Impact of ther COVID-19 Pandemic on Controlling Them

This brief report presents the epidemiological transition of measles and rubella in Japan in 2016–2020. While there was a challenging timeline for eliminating measles in the Western Pacific Region [28], Japan was finally verified as having achieved measles elimination in 2015 [26]. Afterwards, however, several measles outbreaks originating in imported cases were reported [20,21,22,25,26,27]. In 2019, a measles outbreak occurred that stemmed from a reluctance to be vaccinated for religious reasons, implying the importance of considering heterogeneity when implementing vaccination policy [14]. Regarding rubella, Japan experienced two large epidemics in the 2010s. Between 2012 and 2014, the number of rubella patients totaled over 12,000, with 45 cases of congenital rubella syndrome (CRS) [29]. While a supplementary immunization campaign for susceptible pockets was needed, mass vaccination was not implemented because of some operational issues [24]. Before advancing towards the elimination of rubella based on the scientific evidence [29,30,31], Japan again experienced another rubella epidemic in 2018–2020, and 5 CRS cases have been reported [14,29,32].

This brief report also presents a significant decrease in the number of reported cases of measles and rubella in 2020, suggesting that the transmission dynamics of both diseases were occluded. While the supplementary vaccination campaign targeting the rubella epidemic that started in December 2018 might have partly contributed to this decrease, we argue that there were several potential reasons for this achievement, brought about by the COVID-19 pandemic. First, enhanced hygiene measures such as cough etiquette and regular mask wearing [12,13] decreased the transmission of measles and rubella. An online survey revealed that more than 70% of Japanese citizens were accustomed to cough etiquette and mask wearing in late March [12]. Because Japanese citizens were already accustomed to wearing masks to prevent catching and spreading a cold in the winter, and to reduce hay fever in the spring, masks were easily accepted in society, and universal mask use was maintained at a high level of over 80%, even in the summer season [33]. Second, the increasing implementation of work from home (i.e., teleworking), which became more common in response to the COVID-19 pandemic [34], might have positively contributed to interrupting the transmission dynamics of both diseases—especially rubella. While significant advancements in the disease burdens of VPDs have been globally acknowledged among children under five years of age, Japan has faced additional challenges—that is, measles and rubella in adolescence and adulthood. For example, a measles outbreak in Yamagata in 2017 was fueled by young people [25]. In Okinawa in 2018, more than 50% of measles patients were in their 20s or 30s [20]. Susceptible pockets of rubella in adults were also clarified [29]. Considering these susceptible populations, it could be assumed that decreasing human mobility and social contacts in workplaces because of the COVID-19 pandemic could help to decrease the number of VPD patients to some extent. Finally, the significant decline in visitors to Japan also contributed to the decrease in the importation of VPDs. As Japan has vigorously promoted inbound tourism, the number of international visitors to Japan has increased from 8.61 million in 2010 to 31.9 million in 2019 [35]. In line with this, analyzing the risk of importing infectious diseases and ensuring patients’ access to health care have been of great importance [36,37,38,39]. In fact, the rubella epidemic in 2018–2020 was assumed to be triggered by imported cases whose contact history was classified as “unspecified/unknown” [40]. Starting in February 2020, Japan has gradually tightened travel restrictions [7], and the number of visitors in 2020 was limited to 3.97 million as of the end of September [35]. Especially from April to August 2020, the number of international visitors was limited to fewer than 10,000 per month [35], and this huge decrease in international visitors largely contributed to preventing the importation of VPDs.

While these factors could help to contain both diseases in the spring and summer of 2020, it must be acknowledged that the COVID-19 pandemic impeded the maintenance of essential health services in Japan [5,7]. The avoidance of healthcare, which has become especially evident since March 2020 [8], might have caused the underreporting of measles and rubella cases, compared to the pre-pandemic period. In addition, physical distancing mandates have been gradually lifted, and the number of social contacts has started to increase in Japan [41]. As Japan gradually starts to ease the travel restrictions, the importation of infectious diseases can be recognized as an upcoming challenge. While strengthening the surveillance system for imported infectious diseases will be crucial, achieving early detection at ports of entry will be challenging because there are limited resources. The mandatory submission of immunization certificates can be a policy choice, but comprehensive discussion of its effectiveness and issues of human rights will be necessary. Considering a large number of domestically infected cases in Japan with susceptible populations, domestic efforts for containing measles and rubella must not be downgraded.

### 4.2. Challenges in Closing the Gap of Child Vaccination

In the early phase of the COVID-19 pandemic, healthcare resources were largely focused on COVID-19, and essential health services were interrupted in many countries. Routine immunizations were no exception [42]. The United States saw a dramatic decrease in routine vaccines ordered and administered for children after the declaration of a national emergency in March, and lower vaccination coverage among infants and children was reported [43,44,45]. In the United Kingdom, counts of measles, mumps, and rubella (MMR) vaccination fell after the introduction of physical distancing in March [46]. Japan was no exception, and the healthcare system in Japan was stretched to its limits [5,7]. Public health centers, which historically work as the central core of health promotion campaigns, were overwhelmed by their duties for the COVID-19 response [5,7]. Weak health communication brought unreasonable prejudice against healthcare facilities and healthcare providers [5,8]. This made citizens refrain from visiting healthcare institutions and caused a decrease in routine vaccination in Japan.

For example, the survey based on the registered data in the Vaccine Schedules app clarified that the immunization coverage of the first dose of the measles and rubella (MR) vaccine, which is scheduled to be administered at 12–24 months old, has dropped to less than 65% among children born in December 2018 and January 2019 [47]. Similarly, the coverage of the first dose of *Pneumococcal conjugate* vaccine (PCV13), one of the first shots scheduled when a baby is two months old in Japan, was over 90% among children born between April 2018 and September 2019; however, it has decreased to less than 80% among those born in December 2019 and January 2020 [47]. These findings suggest that routine childhood immunization has been disrupted because of the COVID-19 pandemic in Japan. Furthermore, the Japanese Pediatric Society presented data in June showing that the second dose of the MR vaccine, which is scheduled at 5–6 years old, significantly decreased in February and March 2020 compared to the numbers between 2016 and 2019 [48].

The ongoing COVID-19 pandemic starkly reminded us of the importance of vaccines and suggested that the routine immunization system functions under stable conditions, but this disruption of routine childhood vaccination will contribute to the increased risk of measles and rubella outbreaks [43]. An in-person visit is required for immunization services, but concern about COVID-19 easily creates fear among citizens [8], so the risk of their outbreaks will only be minimized after the COVID-19 pandemic is contained. At the same time, both the national government and local governments, along with professional societies, must periodically assess their vaccination coverage, ensure the fair allocation of vaccines depending on the data, promote digital tools for reminders, and develop educational tools for both providers and parents [43].

### 4.3. Time to Invigorate Adult Immunization

While the importance of childhood immunization has been globally acknowledged, and catch-up vaccination campaigns for the younger generation were previously discussed, scant discussion has addressed adult immunization. As noted, measles in adolescence and rubella in middle-aged males have been acknowledged as susceptible populations in Japan; these vulnerabilities are a result of several changes in vaccination policy [14]. However, the progress of supplementary vaccination campaigns has been sluggish [14], partly due to the weakness of health communication that was exposed as a critical challenge during the ongoing COVID-19 response [5,7,8], and also because of insufficient political will to achieve the elimination of rubella [30].

Global attention has been paid to the disruption of childhood immunization campaigns; however, our brief report suggests that the emergence of VPD outbreaks among susceptible adults must be acknowledged as potential health threats, even in high-income countries. Considering that the next Olympic and Paralympic Games are currently scheduled from July to September 2021, Japan must be responsible about preventing the exportation and subsequent outbreaks of VPDs [49]. While the Government of Japan recently launched the coordination meeting for COVID-19 countermeasures at the Tokyo Olympic and Paralympic Games in 2021 [50], it will be necessary to conduct and present a comprehensive health risk assessment, and COVID-19 should not be viewed as the only threat in the preparatory phase of mass-gathering events. To invigorate the ongoing supplementary rubella vaccination campaigns, ensuring opportunities to vaccinate both rubella and seasonal flu shot can be considered as a policy option.

### 4.4. A Window of Opportunity for Science-Based Vaccination Policy and International Cooperation

The COVID-19 pandemic has moved the Overton window for reflecting the latest scientific evidence on advancing public policy at a global scale [51]. It has also created opportunities to review and reflect on lessons learned from previous challenges in vaccination policy in any country, and keenly presented many essential health services that will not be completed by telemedicine. While an appreciation for vaccines is paramount now more than ever, all countries—regardless of their previous investment in VPDs in peacetime—are facing difficulties with administering vaccines at the appropriate time, and innovative catch-up immunization campaigns will be required so that previous elimination efforts are not lost. Experiences in one country or region can be helpful to mitigate the disease burden of VPDs, and they must be swiftly shared as scientific evidence.

In this context, Japan has been strongly committed to global health, accelerated universal health coverage, and expedited global health security [52,53,54]; however, these efforts must be aligned with domestic efforts to contain measles and rubella. Vaccine hesitancy in Japan has been acknowledged as a significant challenge at the global level [55], and must be earnestly tackled by strengthening health communication. A discrepancy between Japan’s global commitment in the VPDs measles and rubella and domestic vaccination policy [56,57], weaknesses in governance [58], and politicization [59] has been previously argued; however, the ongoing supplementary immunization campaign for rubella epidemic does not fully reflect the latest evidence [29,31,60]. It should be noted that a rubella epidemic has impacted demography [61] and must be conceptualized in the context of improving population health in Japan. A human security approach—a central concept of Japan’s foreign policy that will also be pivotal in the ongoing COVID-19 response [6,52,53,54]—should be applied to citizens in Japan. Reviewing the evolution of the previous vaccination policy, reflecting on lessons learned through an evidence-based approach, and showcasing Japan’s domestic efforts will help strengthen regional health security and reinforce Japan’s commitment to eliminating VPDs. To complete these processes, coordination between healthcare workers, government officials, and civil society, along with high political commitments, will be pivotal.

## 5. Conclusions

This brief report summarizes the epidemiological trends of measles and rubella in Japan from 2016 to 2020. While the transmission of measles and rubella seems to be suppressed during the COVID-19 pandemic, susceptible populations of measles and rubella in Japan must be acknowledged in order to avoid the resurgence of these diseases. Ensuring citizens’ access to vaccines and invigorating the ongoing supplementary vaccination campaigns will be vital during and after the COVID-19 pandemic. To what extent the COVID-19 pandemic impacted efforts to break chains of transmissions of measles and rubella needs to be analyzed by multiple approaches, including the quasi-experimental design, which is our future research topic. Scientists, healthcare workers, policymakers, and civil society must utilize a window of opportunity brought by the COVID-19 pandemic to revamp the previously politicized vaccination policy and incorporate the latest evidence.

## Figures and Tables

**Table 1:** Reported measles cases per million, by prefecture [16,17,19].

Place/Year	2016	2017	2018	2019	2020 *
Tokyo	1.7	2.1	1.6	9.2	0.1
Japan	1.2	1.5	2.2	5.9	0.1

* As of 30 September (Week 39), 2020. (Publicly available data. No copyright issues).

**Table 2:** Reported rubella cases per million, by prefecture [16,18,19].

Place/Year	2016	2017	2018	2019	2020 *
Tokyo	1.4	1.6	69.9	63.2	1.7
Japan	1.0	0.7	22.9	18.1	0.7

* As of 30 September (Week 39), 2020. (Publicly available data. No copyright issues).

**Table 3:** Reported measles cases, aggregated by acquired regions [16,17,19].

Acquired Region/Year	2016	2017	2018	2019	2020 *
Domestic	123 (77.4%)	155 (82.0%)	217 (77.0%)	558 (75.0%)	7 (58.3%)
Imported	27 (17.0%)	31 (16.4%)	34 (12.1%)	110 (14.8%)	3 (25.0%)
Unspecified/Unknown	9 (5.66%)	3 (1.59%)	31 (11.0%)	76 (10.2%)	2 (16.7%)
Total	159	189	282	744	12

* As of 30 September (Week 39), 2020. (Publicly available data. No copyright issues).

**Table 4:** Reported rubella cases, aggregated by acquired region [16,18,19].

Acquired Region/Year	2016	2017	2018	2019	2020 *
Domestic	114 (91.2%)	78 (83.9%)	2251 (77.2%)	1783 (77.3%)	59 (65.6%)
Imported	9 (7.20%)	11 (11.8%)	21 (0.72%)	50 (2.17%)	13 (14.4%)
Unspecified/Unknown	2 (1.60%)	4 (4.3%)	645 (22.1%)	473 (20.5%)	18 (20.0%)
Total	125	93	2917	2306	90

* As of 30 September (Week 39), 2020. (Publicly available data. No copyright issues).

**Table 5:** Reported measles cases, aggregated by age and vaccination history [16,17,19].

Characteristics	Group	2016	2017	2018	2019	2020 *
Age (years)	0–9	31 (19.5%)	16 (8.5%)	51 (18.1%)	112 (15.1%)	5 (41.7%)
	10–19	14 (8.8%)	20 (10.6%)	28 (9.9%)	109 (14.7%)	1 (8.3%)
	20–29	57 (35.8%)	63 (33.3%)	86 (30.5%)	198 (26.6%)	1 (8.3%)
	30–39	34 (21.4%)	61 (32.3%)	73 (25.9%)	216 (29.0%)	2 (16.7%)
	40–49	14 (8.8%)	21 (11.1%)	32 (11.3%)	87 (11.7%)	2 (16.7%)
	50+	9 (5.7%)	8 (4.2%)	12 (4.3%)	22 (3.0%)	1 (8.3%)
Vaccination history	1+ doses	63 (39.6%)	70 (37.0%)	99 (35.1%)	264 (35.5%)	7 (58.3%)
	0 doses	43 (27.0%)	34 (18.0%)	63 (22.3%)	194 (26.1%)	1 (8.3%)
	Unknown	53 (33.3%)	85 (45.0%)	120 (42.6%)	286 (38.4%)	4 (33.3%)
Total		159	189	282	744	12

* As of 30 September (Week 39), 2020. (Publicly available data. No copyright issues).

**Table 6:** Reported rubella cases, aggregated by age and vaccination history [16,18,19].

Characteristics	Sex	Group	2016	2017	2018	2019	2020 *
Age (years)	Male	0–9	10 (8.0%)	12 (12.9%)	20 (0.7%)	23 (1.0%)	4 (4.4%)
		10–19	5 (4.0%)	9 (9.7%)	43 (1.5%)	46 (2.0%)	4 (4.4%)
		20–29	15 (12.0%)	6 (6.5%)	355 (12.2%)	370 (16.0%)	10 (11.1%)
		30–39	17 (13.6%)	10 (10.8%)	616 (21.1%)	456 (19.8%)	9 (10.0%)
		40–49	14 (11.2%)	9 (9.7%)	878 (30.1%)	617 (26.8%)	24 (26.7%)
		50–59	6 (4.8%)	11 (11.8%)	391 (13.4%)	234 (10.1%)	10 (11.1%)
		60+	6 (4.8%)	3 (3.2%)	61 (2.1%)	58 (2.5%)	4 (4.4%)
	Female	0–9	13 (10.4%)	7 (7.5%)	16 (0.5%)	20 (0.9%)	2 (2.2%)
		10–19	2 (1.6%)	2 (2.2%)	46 (1.6%)	41 (1.8%)	3 (3.3%)
		20–29	15 (12.0%)	10 (10.8%)	191 (6.5%)	175 (7.6%)	7 (7.8%)
		30–39	8 (6.4%)	6 (6.5%)	142 (4.9%)	145 (6.3%)	6 (6.7%)
		40–49	8 (6.4%)	1 (1.1%)	70 (2.4%)	63 (2.7%)	3 (3.3%)
		50–59	3 (2.4%)	5 (5.4%)	61 (2.1%)	39 (1.7%)	2 (2.2%)
		60+	3 (2.4%)	2 (2.2%)	27 (0.9%)	19 (0.8%)	2 (2.2%)
Vaccination history	Male	1+ doses	14 (11.2%)	17 (18.3%)	119 (4.1%)	132 (5.7%)	12 (13.3%)
		0 doses	16 (12.8%)	9 (9.7%)	615 (21.1%)	389 (16.9%)	14 (15.6%)
		Unknown	43 (34.4%)	34 (36.6%)	1630 (55.9%)	1283 (55.6%)	39 (43.3%)
	Female	1+ doses	19 (15.2%)	9 (9.7%)	74 (2.5%)	92 (4.0%)	5 (5.6%)
		0 doses	8 (6.4%)	8 (8.6%)	140 (4.8%)	90 (3.9%)	2 (2.2%)
		Unknown	25 (20.0%)	16 (17.2%)	339 (11.6%)	320 (13.9%)	18 (20.0%)
Total			125	93	2917	2306	90

* As of 30 September (Week 39), 2020. (Publicly available data. No copyright issues).

## References

[1] Hanna T.P., Evans G.A., Booth C.M. (2020). Cancer, COVID-19 and the Precautionary Principle: Prioritizing Treatment during a Global Pandemic. Nat. Rev. Clin. Oncol..

[2] (2020). COVIDSurg Collaborative. Elective Surgery Cancellations Due to the COVID-19 Pandemic: Global Predictive Modelling to Inform Surgical Recovery Plans. Br. J. Surg..

[3] Abbas K., Procter S.R., van Zandvoort K., Clark A., Funk S., Mengistu T., Hogan D., Dansereau E., Jit M., Flasche S. (2020). Routine Childhood Immunisation during the COVID-19 Pandemic in Africa: A Benefit–Risk Analysis of Health Benefits versus Excess Risk of SARS-CoV-2 Infection. Lancet Glob. Health.

[4] Nelson B. (2020). The Positive Effects of Covid-19. BMJ.

[5] Shimizu K., Wharton G., Sakamoto H., Mossialos E. (2020). Resurgence of Covid-19 in Japan. BMJ.

[6] Shimizu K., Kondo T., Tokuda Y., Shibuya K. (2020). An Open Letter to Japan’s New Prime Minister. Lancet.

[7] Shimizu K., Negita M. (2020). Lessons Learned from Japan’s Response to the First Wave of COVID-19: A Content Analysis. Healthcare.

[8] Shimizu K., Lin L. (2020). Defamation against Healthcare Workers During COVID-19 Pandemic. Int. J. Health Policy Manag..

[9] Shimizu K., Mossialos E. (2020). Accountability and transparency are vital in a pandemic response. J. Gen. Fam. Med..

[10] Legido-Quigley H., Asgari N., Teo Y.Y., Leung G.M., Oshitani H., Fukuda K., Cook A.R., Hsu L.Y., Shibuya K., Heymann D. (2020). Are High-Performing Health Systems Resilient against the COVID-19 Epidemic?. Lancet.

[11] Han E., Tan M.M.J., Turk E., Sridhar D., Leung G.M., Shibuya K., Asgari N., Oh J., García-Basteiro A.L., Hanefeld J. (2020). Lessons Learnt from Easing COVID-19 Restrictions: An Analysis of Countries and Regions in Asia Pacific and Europe. Lancet.

[12] Muto K., Yamamoto I., Nagasu M., Tanaka M., Wada K. (2020). Japanese Citizens’ Behavioral Changes and Preparedness against COVID-19: An Online Survey during the Early Phase of the Pandemic. PLoS ONE.

[13] Nomura S., Yoneoka D., Tanoue Y., Kawashima T., Shi S., Eguchi A., Miyata H. (2020). Time to Reconsider Diverse Ways of Working in Japan to Promote Social Distancing Measures against the COVID-19. J. Urban Health.

[14] Shimizu K., Sorano S., Iwai K. (2020). Vaccine Hesitancy in Japan: Is the Country Well Prepared for Tokyo 2020?. Travel Med. Infect. Dis..

[15] Nakamura S., Wada K., Yanagisawa N., Smith D.R. (2018). Health Risks and Precautions for Visitors to the Tokyo 2020 Olympic and Paralympic Games. Travel Med. Infect. Dis..

[16] National Institute of Infectious Diseases (NIID) Infectious Diseases Weekly Report (IDWR). https://www.niid.go.jp/niid/en/idwr-e.html.

[17] NIID. IDWR. Measles. https://www.niid.go.jp/niid/ja/hassei/575-measles-doko.html.

[18] NIID. IDWR. Rubella. IDWR. https://www.niid.go.jp/niid/ja/rubella-m-111/rubella-top/700-idsc/2131-rubella-doko.html.

[19] Tokyo Metropolitan Infectious Disease Surveillance Center National Epidemiological Surveillance of Infectious Diseases in Tokyo. https://survey.tokyo-eiken.go.jp/epidinfo/weeklyzensu.do.

[20] Shimizu K., Kinoshita R., Yoshii K., Akhmetzhanov A., Jung S., Lee H., Nishiura H. (2018). An Investigation of a Measles Outbreak in Japan and Taiwan, China, March–May 2018. West. Pacific Surveill. Response J..

[21] Akhmetzhanov A.R., Lee H., Jung S., Kinoshita R., Shimizu K., Yoshii K., Nishiura H. (2018). Real Time Forecasting of Measles Using Generation-Dependent Mathematical Model in Japan. PLoS Curr..

[22] Mizumoto K., Kobayashi T., Chowell G. (2018). Transmission Potential of Modified Measles during an Outbreak, Japan, March‒May 2018. Euro Surveill..

[23] Tanaka-Taya K., Satoh H., Arai S., Yamagishi T., Yahata Y., Nakashima K., Sugawara T., Ohkusa Y., Matsui T., Saito T. (2013). Nationwide Rubella Epidemic-Japan, 2013. MMWR. Morb. Mortal. Wkly. Rep..

[24] Ujiie M., Nabae K., Shobayashi T. (2014). Rubella Outbreak in Japan. Lancet.

[25] Komabayashi K., Seto J., Tanaka S., Suzuki Y., Ikeda T., Onuki N., Yamada K., Ahiko T., Ishikawa H., Mizuta K. (2018). The Largest Measles Outbreak, Including 38 Modified Measles and 22 Typical Measles Cases in Its Elimination Era in Yamagata, Japan, 2017. Jpn. J. Infect. Dis..

[26] Kinoshita R., Shimizu K., Nishiura H. (2018). Measles Control in a Measles-Eliminated Country, Japan. Travel Med. Infect. Dis..

[27] Nishiura H., Mizumoto K., Asai Y. (2017). Assessing the Transmission Dynamics of Measles in Japan, 2016. Epidemics.

[28] Masuno K., Shibuya K. (2009). Measles Elimination: Lack of Progress in the Western Pacific Region. Lancet.

[29] Kayano T., Lee H., Nishiura H. (2019). Modelling a Supplementary Vaccination Program of Rubella Using the 2012-2013 Epidemic Data in Japan. Int. J. Environ. Res. Public Health.

[30] Jindai K., Funaki T., Nishijima T., Takakura S., Noda H., Miyake K. (2018). Towards Rubella Elimination in Japan. Lancet Infect. Dis..

[31] Saito M.M., Ejima K., Kinoshita R., Nishiura H. (2018). Assessing the Effectiveness and Cost-Benefit of Test-and-Vaccinate Policy for Supplementary Vaccination against Rubella with Limited Doses. Int. J. Environ. Res. Public Health.

[32] Lee H., Kayano T., Nishiura H. (2019). Predicting Congenital Rubella Syndrome in Japan, 2018–2019. Int. J. Infect. Dis..

[33] Institute for Health Metrics and Evaluation COVID-19 Projections. Mask Use. https://covid19.healthdata.org/japan?view=mask-use&tab=trend.

[34] Eguchi A., Yoneoka D., Shi S., Tanoue Y., Kawashima T., Nomura S., Matsuura K., Makiyama K., Ejima K., Gilmour S. (2020). Trend Change of the Transmission Route of COVID-19–Related Symptoms in Japan. Public Health.

[35] Japan National Tourism Organization Number of Inbound and Outbound Travelers. https://www.jnto.go.jp/jpn/statistics/since2003_visitor_arrivals.xlsx.

[36] Anzai A., Kawatsu L., Uchimura K., Nishiura H. (2020). Reconstructing the Population Dynamics of Foreign Residents in Japan to Estimate the Prevalence of Infection with Mycobacterium Tuberculosis. J. Theor. Biol..

[37] Yuan B., Nishiura H. (2018). Estimating the Actual Importation Risk of Dengue Virus Infection among Japanese Travelers. PLoS ONE.

[38] Yuan B., Lee H., Nishiura H. (2019). Assessing Dengue Control in Tokyo, 2014. PLoS Negl. Trop. Dis..

[39] Shimizu K., Nishiura H., Imamura A. (2019). Investigation of the Proportion of Diagnosed People Living with HIV/AIDS among Foreign Residents in Japan. J. Clin. Med..

[40] Nishiura H., Kayano T., Kinoshita R. (2019). Overcoming the Difficulty of Achieving Elimination Status for Measles and Rubella Due to Imported Infections: Estimation of the Reproduction Number R for Measles and Rubella. Travel Med. Infect. Dis..

[41] Institute for Health Metrics and Evaluation COVID-19 Projections. Social Distancing. https://covid19.healthdata.org/japan?view=social-distancing&tab=trend.

[42] Hungerford D., Cunliffe N.A. (2020). Coronavirus Disease (COVID-19)-Impact on Vaccine Preventable Diseases. Euro Surveill..

[43] Santoli J.M., Lindley M.C., DeSilva M.B., Kharbanda E.O., Daley M.F., Galloway L., Gee J., Glover M., Herring B., Kang Y. (2020). Effects of the COVID-19 Pandemic on Routine Pediatric Vaccine Ordering and Administration—United States, 2020. MMWR. Morb. Mortal. Wkly. Rep..

[44] Bramer C.A., Kimmins L.M., Swanson R., Kuo J., Vranesich P., Jacques-Carroll L.A., Shen A.K. (2020). Decline in Child Vaccination Coverage During the COVID-19 Pandemic—Michigan Care Improvement Registry, May 2016–May 2020. MMWR. Morb. Mortal. Wkly. Rep..

[45] Stephenson J. (2020). Sharp Drop in Routine Vaccinations for US Children amid COVID-19 Pandemic. JAMA Health Forum.

[46] McDonald H.I., Tessier E., White J.M., Woodruff M., Knowles C., Bates C., Parry J., Walker J.L., Scott J.A., Smeeth L. (2020). Early Impact of the Coronavirus Disease (COVID-19) Pandemic and Physical Distancing Measures on Routine Childhood Vaccinations in England, January to April 2020. Euro Surveill..

[47] Know VPD. Press Release: The Immunization Coverage among Children Has Decreased due to COVID-19 Pandemic. https://www.know-vpd.jp/news/20741.php.

[48] Japan Pediatric Society Childhood Immunization under the COVID-19 Pandemic. http://www.jpeds.or.jp/uploads/files/20200617_yobosesshu.pdf.

[49] Shimizu K., Devoid I. The 2020 Olympics and Paralympic Games & COVID-19. https://blogs.bmj.com/bmjgh/2020/05/07/the-2020-olympics-and-paralympic-games-covid-19/.

[50] The Tokyo Organising Committee of the Olympic and Paralympic Games First Coordination Meeting for COVID-19 Countermeasures at the Olympic and Paralympic Games Tokyo 2020. https://tokyo2020.org/en/news/first-coordination-meeting-for-covid-19-countermeasures-at-tokyo2020.

[51] Golembeski C., Irfan A., Williams B., Venters H. COVID-19 amidst Carceral Contexts: The Overtone Window of Political Possibility and Policy Change. https://jphmpdirect.com/2020/04/20/covid-19-amidst-carceral-contexts-the-overton-window-of-political-possibility-and-policy-change/.

[52] Shibuya K., Nomura S., Okayasu H., Ezoe S., Hara S., Hara Y., Izutsu T., Kato T., Mabuchi S., Maeda Y. (2016). Protecting Human Security: Proposals for the G7 Ise-Shima Summit in Japan. Lancet.

[53] Abe S. (2013). Japan’s Strategy for Global Health Diplomacy: Why It Matters. Lancet.

[54] Abe S. (2015). Japan’s Vision for a Peaceful and Healthier World. Lancet.

[55] De Figueiredo A., Simas C., Karafillakis E., Paterson P., Larson H.J. (2020). Mapping Global Trends in Vaccine Confidence and Investigating Barriers to Vaccine Uptake: A Large-Scale Retrospective Temporal Modelling Study. Lancet.

[56] Murashige N., Matsumura T., Masahiro K. (2011). Disseminating Japan’s Immunisation Policy to the World. Lancet.

[57] Hosoda M., Inoue H., Miyazawa Y., Kusumi E., Shibuya K. (2012). Vaccine-Associated Paralytic Poliomyelitis in Japan. Lancet.

[58] Gilmour S., Kanda M., Kusumi E., Tanimoto T., Kami M., Shibuya K. (2013). HPV Vaccination Programme in Japan. Lancet.

[59] Tanimoto T., Murashige N., Hosoda M., Kusumi E., Ono S., Kami M., Shibuya K. (2012). Vaccination for Whom? Time to Reinvigorate Japanese Vaccine Policy. Lancet.

[60] Kayano T., Lee H., Kinoshita R., Nishiura H. (2020). Identifying Geographic Areas at Risk of Rubella Epidemics in Japan Using Seroepidemiological Data. Int. J. Infect. Dis..

[61] Mizumoto K., Chowell G. (2020). Temporary Fertility Decline after Large Rubella Outbreak, Japan. Emerg. Infect. Dis..

